# Dietary perceptions and challenges in the management of type 2 diabetes in rural communities of the Eastern Cape, South Africa: a qualitative study

**DOI:** 10.3389/fcdhc.2026.1835644

**Published:** 2026-07-08

**Authors:** Ncebakazi Nkaule, Ntiyiso Vinny Khosa, Ziphelele Ncane, Sibusio Cyprian Nomatshila

**Affiliations:** 1 School of Public Health, Faculty of Medicine and Health Sciences, iYunivesithi Walter Sisulu, Mthatha, South Africa; 2 Waler Sisulu Society and Health Research Institute, Faculty of Medicine and Health Sciences, iYunivesithi Walter Sisulu, Mthatha, South Africa

**Keywords:** diabetes mellitus, dietary barriers, dietary behaviour, healthy eating, rural communities

## Abstract

Diabetes mellitus (DM) is an increasing public health concern globally, particularly in low- and middle-income countries (LMICs). Socioeconomic challenges complicate disease management. Dietary management is central to diabetes care; however, adherence to recommended dietary practices remains difficult in many rural and resource-constrained settings. This study explored perceptions of healthy eating and barriers influencing dietary practices among individuals living with diabetes in rural communities of the Mnquma sub-region in the Eastern Cape (EC), South Africa (SA). A qualitative descriptive study was conducted among adults diagnosed with type 2 diabetes attending four primary healthcare clinics. Purposive sampling was used to recruit 35 participants. Data were collected through semi-structured interviews conducted in IsiXhosa or English, audio-recorded, transcribed verbatim, translated into English, and analysed using thematic analysis. Four major themes and fifteen sub-themes emerged: knowledge and perceptions of diabetes and healthy eating; emotional and psychological factors affecting eating habits; structural, economic, and environmental barriers to healthy eating; and the role of healthcare providers and support systems. Understanding of diabetes and healthy eating, with dietary practices often shaped by personal experiences rather than formal health education. Emotional responses to diagnosis, including fear and distress, influenced self-management behaviours. Financial insecurity, food insecurity, household food practices, agricultural instability, competing health conditions, and inconsistent dietary information emerged as major barriers to healthy eating. Although healthcare professionals were viewed as trusted sources of information, participants reported limited dietary counselling and fragmented health messages, while family support varied across households. These findings highlight that healthy eating among individuals living with diabetes in rural Mnquma is shaped by complex interactions between knowledge, emotional experiences, socioeconomic conditions, and health system support. While consistent with the Theory of Planned Behaviour (TPB), the results also highlight the importance of broader socioecological influences in shaping behaviour in resource-constrained settings. These findings underscore the need for context-sensitive diabetes interventions that extend beyond individual-level education to strengthen family and community support, improve dietary counselling within primary healthcare, and address broader socioecological and structural determinants, including food insecurity, poverty, and constrained food environments in rural settings.

## 1 Introduction

Diabetes mellitus (DM) is a chronic metabolic disorder characterised by persistent hyperglycaemia resulting from defects in insulin secretion, insulin action, or both (1). Diabetes is broadly classified into type 1 diabetes, an autoimmune condition characterised by absolute insulin deficiency, and type 2 diabetes mellitus (T2DM), which accounts for the majority of cases globally and is strongly associated with behavioural, environmental and metabolic risk factors (2)

The global burden of diabetes has increased substantially in recent decades, making it one of the most pressing public health challenges of the 21st century. It is estimated that approximately one in eleven adults worldwide currently live with diabetes, with projections indicating that the number of affected individuals may reach nearly 693 million by 2045 (3). Diabetes prevalence in sub-Saharan Africa is increasing rapidly, with approximately 24.6 million adults currently living with diabetes, and projections indicating a rise to nearly 60 million by 2050 (4). South Africa (SA) reflects these global trends. In SA, diabetes is a major and growing public health concern, with an estimated 4.2 million adults living with the condition (4). It is also among the leading causes of death in the country, underscoring its significant health burden (5). The rising prevalence places significant pressure on the public healthcare system, particularly in rural and resource-constrained provinces such as the Eastern Cape (EC). Data from the Eastern Cape Province, which includes rural districts such as O.R. Tambo and parts of the former Transkei and Ciskei homelands, indicate a growing incidence of diabetes in recent years, closely aligned with overall national increases (6).

The surge in diabetes prevalence globally and within SA is linked to modifiable risk factors, especially those related to diet and physical activity patterns (7). Modern lifestyles increasingly expose populations to energy-dense, nutrient-poor foods, limited opportunities for physical activity, and economic environments that constrain healthy choices (8). Many individuals in rural and low-income communities have limited or incomplete knowledge about the causes and management of diabetes (9). A comparative analysis conducted in Nigeria and South Africa demonstrated a strong correlation between diabetes prevalence and high dependence on complementary and alternative medicine, the cost of diabetes treatment, limited health system capacity, and poor adherence to diabetes management guidelines (10). Limited knowledge about the role of diet often results in poor adherence to dietary recommendations, despite clinical advice (11).

DM presents a growing global health challenge, driven by dietary shifts, lifestyle changes, and socioeconomic conditions (12). In SA, and particularly in the EC’s rural communities, rising prevalence and limited diabetes knowledge are compounded by barriers to healthy eating and lifestyle adaptation (13). While the influence of social determinants such as poverty, food insecurity, and limitations in the health system on diabetes management is well established, there is limited qualitative research exploring how individuals in rural South African contexts interpret and navigate dietary recommendations within these constraint (14). Existing studies often focus on knowledge deficits or adherence outcomes, with less attention given to the lived experiences of individuals managing diabetes within complex household, cultural, and economic environments (14). In particular, there is a lack of context-specific evidence from rural areas of the EC, where structural inequalities, food insecurity, and limited access to dietetic services intersect to influence dietary behaviours. This study therefore seeks to address this gap by exploring how individuals living with diabetes in the Mnquma sub-region perceive healthy eating and experience barriers to dietary adherence within their everyday lives.

## 2 Materials and methods

### 2.1 Theoretical framework

This study was guided by the Theory of Planned Behaviour (TPB), which provides a framework for understanding health-related behaviours, including dietary practices among individuals living with diabetes (15). According to TPB, behaviour is influenced by three key constructs: attitudes towards behaviour, subjective norms (social influences), and perceived behavioural control.

The TPB was considered appropriate for this study as it enabled exploration of how individual beliefs, social pressures, and perceived barriers influence dietary practices in resource-constrained settings. These constructs informed both the development of the interview guide and the interpretation of findings, particularly in understanding how knowledge, social support, and structural challenges shape healthy eating behaviours among participants. While the TPB guided the exploration of individual-level determinants of dietary behaviour, the analysis remained open to broader contextual influences emerging from participants’ experiences.

### 2.2 Study design

This study employed a qualitative descriptive research approach to explore complex, context-specific issues such as individual beliefs, perceptions, and behaviours. A qualitative descriptive approach was considered appropriate as it enables the researcher to gather rich, in-depth data on how participants understand and experience their world (16). This approach was appropriate for gaining in-depth insights into participants lived experiences and understanding contextual factors influencing dietary behaviours within rural communities.

### 2.3 Study setting

The study was conducted in the Mnquma sub-region of the EC Province, SA. Participants were recruited from four primary healthcare clinics that provide chronic disease management services: Butterworth Gateway Clinic, Ngqamakhwe Clinic, Nozuko Clinic in Msobomvu Township, and Ibika Clinic. These facilities serve rural and peri-urban populations characterised by high levels of socioeconomic disadvantage and limited access to specialised healthcare services, including dietetic support.

### 2.4 Participants and sampling

Participants were adults aged 18 years and older who had been diagnosed with T2DM and were receiving care at the selected primary healthcare clinics. Purposive sampling was used to recruit participants with lived experience of diabetes to capture diverse perspectives across age, gender, clinic site, and socioeconomic circumstances, as reflected in employment status and reliance on social grants. While efforts were made to include diverse participants, recruitment was influenced by clinic attendance patterns, which may have contributed to the predominance of female participants. A total of 35 participants were recruited and participated in the study. Data saturation was assessed as the point at which no new themes or meaningful insights were emerging from the data, consistent with established qualitative research principles (17). In this study, saturation was reached at participant 33, after which two additional interviews were conducted to confirm that no new information was identified. The study aimed to achieve data saturation rather than theoretical saturation, consistent with a qualitative descriptive approach.

### 2.5 Data collection

Data were collected through semi-structured face-to-face interviews conducted in a private space within the health facilities premises to ensure confidentiality. Interviews were conducted in either IsiXhosa or English according to participant preference. All interviews were audio-recorded after receipt of participants’ consent and they lasted between 10–20 minutes, however depth was achieved through the use of a focused semi-structured interview guide with open-ended questions, supported by probing and follow-up questions that allowed participants to elaborate on their experiences. In addition, participants were purposively selected based on their lived experience of diabetes management, which facilitated the generation of rich, relevant, and context-specific accounts. Data collection continued until data saturation was reached, with no new themes emerging across successive interviews. The interview guide was informed by the construction of the TPB and is provided as **Supplementary Material** (**Appendices A**, **B**) (15). Data collection was conducted between August and October 2025.

### 2.6 Data analysis

Interviews were transcribed verbatim by the primary researcher. IsiXhosa transcripts were translated into English primarily by the primary researcher (NN), who is fluent in IsiXhosa, ensuring that contextual meaning and linguistic nuances were accurately preserved. AI-assisted translation tools were used only as a supplementary aid to support initial wording consistency and fluency, and all outputs were critically reviewed and manually corrected by the researcher. To further enhance translation quality, selected transcripts and translated excerpts were cross-checked against the original audio recordings, and discrepancies were discussed and resolved through careful review. In addition, a co-author (SN) experienced in qualitative research independently reviewed selected transcripts to verify meaning, consistency, and accurate representation of participants’ views.

The data were analysed using thematic analysis following the six-phase framework described by Braun and Clarke (18). Transcripts were read repeatedly to ensure familiarity with the data. Coding was conducted manually by the primary researcher (NN) using an inductive approach. Initial codes were generated through line-by-line coding and iteratively refined as patterns emerged across transcripts. A codebook was developed and continuously refined throughout the analysis process, comprising codes, categories, and emerging themes, which were reorganized as analysis progressed. Related codes were then grouped into broader categories. These categories were then developed into themes through constant comparison across transcripts. Themes were continuously reviewed and refined against the coded data and full dataset to ensure coherence and internal consistency.

To enhance analytical rigour, a subset of transcripts and corresponding codes were reviewed in consultation with the co-authors (SN and ZN). Differences in interpretation were discussed until consensus was reached, thereby strengthening the credibility and consistency of the analysis. Reflexive practices were applied throughout the analysis process, including ongoing reflection on the researchers’ assumptions and their potential influence on interpretation. An audit trail was maintained documenting coding decisions, codebook revisions, and theme development throughout the analysis process. The final codebook is provided as **Supplementary Table 1**.

Recommendations by Lincoln and Guba were applied to ensure trustworthiness through credibility, transferability, dependability, and confirmability (19). Credibility was enhanced through prolonged engagement with participants and the use of verbatim quotations to support findings. Transferability was addressed by providing detailed descriptions of the study context and participant characteristics. Dependability was ensured through maintaining an audit trail of the research process, including data collection and analysis decisions. Confirmability was strengthened through reflexive practices and regular discussions among the research team to minimise potential researcher bias.

### 2.7 Reflexivity and positionality

Data collection and initial analysis were conducted by the primary researcher (NN), a dietitian employed at a district hospital within the broader study area. Although the researcher was not directly employed at the participating primary healthcare clinics, familiarity with the local healthcare context and patient population facilitated rapport during data collection. However, this familiarity also introduced the potential for bias. To address this, the researcher engaged in reflexive practices throughout the study, including documenting assumptions and reflecting on interactions during data collection and analysis. Regular discussions were held with co-authors (SN and ZN) experienced in qualitative research to support critical reflection and ensure that findings remained grounded in participants’ perspectives. The researcher remained aware of her professional background and its potential influence on interpretation and actively addressed this through ongoing reflexive journaling, critical self-reflection, and regular discussions with co-authors to challenge assumptions and ensure that findings were grounded in participants’ accounts.

Ethical approval for the study was obtained from the Walter Sisulu University Health Research Ethics Committee (WSU HREC 181/2025). Permission to conduct the study was granted by the Eastern Cape Department of Health and the participating healthcare facilities. Written informed consent was obtained from all participants prior to participation.

## 3 Results

### 3.1 Participant characteristics

A total of 35 participants diagnosed with T2DM participated in the study. The majority were females (n = 32), and most participants (n=18) were aged 51–65 years. The majority (n=27) had completed secondary education, while over half (n=18) were dependent on social grants or pensions as their primary source of income. Participants were recruited from four primary healthcare clinics within the Mnquma sub-region.

As shown in **Table 1**, the sample included participants from four clinics, reflecting a predominantly rural and socio-economically disadvantaged population.

**Table 1:** Demographic characteristics of participants (N = 35).

Characteristic	Category	Frequency (n)
Age (years)	18 – 35	3
	36 – 50	5
	51 – 65	18
	>65	9
Sex	Female	32
	Male	3
Marital Status	Single	11
	Married	14
	Widowed	7
	Separated/Divorced	3
Education Level	Primary (≤ Grade 7)	5
	Secondary (Grade 8–12)	27
	Post-secondary	3
Employment Status	Employed	4
	Unemployed	13
	Pension/Grant dependent	18
Clinic Site	Clinic A	4
	Clinic B	12
	Clinic C	10
	Clinic D	9

### 3.2 Overview of themes

Thematic analysis of the interview data resulted in four major themes and fifteen sub-themes reflecting participants’ perceptions of diabetes, emotional responses to diagnosis, barriers to healthy eating, and the influence of healthcare support systems.

**Table 2** summarises the main themes and sub-themes that emerged from the thematic analysis of the interview data. Each theme is presented and discussed below, with illustrative participant quotations used to demonstrate how the findings address the study objectives.

**Table 2:** Summary of themes and subthemes.

Theme	Sub-themes
Theme 1: Knowledge and perceptions of diabetes and healthy eating	• Limited and fragmented understanding of diabetes • Participants’ perceptions regarding causes of diabetes. • Basic medical knowledge mixed with misunderstandings
Theme 2: Emotional and psychological factors affecting eating habits	• Emotional shock, fear, and distress at diagnosis • Processes of acceptance and adjustment • Psychological burden of poor glycaemic control
Theme 3: Structural, economic, and environmental barriers to healthy eating	• Financial constraints and food insecurity • Household and social eating practices • Agricultural instability and barriers to home food production • Competing health conditions and physical limitations • Knowledge gaps and conflicting dietary information
Theme 4: Role of healthcare providers and support systems	• Trust in healthcare providers • Inadequate dietary counselling • Fragmented and inconsistent health messages • Family and social support dynamics

### 3.3 Theme 1: knowledge and perceptions of diabetes and healthy eating

Participants demonstrated partial and fragmented knowledge of diabetes and its dietary management. Although many recognised the importance of diet, their understanding was often simplified and shaped by personal experience rather than formal health education.

Limited and fragmented understanding of diabetes

More than half of the participants (n = 17) demonstrated limited understanding of diabetes, including uncertainty regarding its causes, management, and long-term implications.

When asked about their understanding of diabetes, some participants expressed confusion or lack of knowledge about the condition.

*“Honestly, I don’t really understand anything clearly.”* (Clinic -A, P03-Female, 36-50)

Others indicated that their understanding was minimal and largely based on hearsay or treatment experience:

*“I do not really have knowledge. I just hear that when a person has diabetes, they are given treatment.”* (Clinic C, P19-Female, 51-65)

*“I do not have knowledge.”* (Clinic D, P27-Female, 51-65)

Some participants explained that they still did not fully understand diabetes despite living with the condition for some time.

*“I do not know what it is, even now I still do not fully understand it.”* (Clinic D, P29-Female, >65)

Some participants indicated that information received at diagnosis was forgotten over time.

*“I’ve forgotten now because I was once told everything.”* (Clinic A, P02-Female, 36-50)

Participants’ perceptions regarding causes of diabetes.

Some participants (n = 19) associated diabetes primarily with dietary factors such as sugar and starch consumption, with limited recognition of the condition’s metabolic or multifactorial nature.

When asked what they believed causes diabetes, participants frequently linked the condition to unhealthy eating habits.

*“I think it’s the diet.”* (Clinic A, P01-Female, 51-65)

*“I think it is caused by eating too much sugar.”* (Clinic B, P12-Female, 51-65)

Some participants also associated diabetes with hereditary factors.

“*It is said to be hereditary*.” (Clinic C, P24-Female, 51-65)

Others attributed it to a range of perceived factors, including heredity, stress, lifestyle behaviours, and dietary practices. Heredity was frequently mentioned, particularly among those with a family history of the condition.

*“I think it is genetic, because my mother had it.”* (Clinic B, P16-Female, 36-50)

*“My father and uncle had diabetes, and they passed away.”* (Clinic D-P31-Female, 18-35)

One participant believed that emotional stress and prolonged unhappiness contributed to the development of diabetes.

*“Too much thinking or stress, unhappiness.”* (Clinic A, P02-Female, 36-50)

Basic medical knowledge mixed with misunderstandings.

A smaller number of participants (n = 7) demonstrated basic biomedical understanding of diabetes, particularly regarding potential complications associated with poor disease control. However, this knowledge appeared to be shaped mainly by personal experiences and community observations rather than formal education.

One participant associated diabetes with kidney complications and physical weakness.

*“Diabetes destroys the kidneys and makes you lose energy.”* (Clinic A, P01-Female, 51-65)

Another participant described awareness of severe complications related to uncontrolled diabetes.

“*Diabetes needs to be controlled because if you don’t control it, you end up having your legs amputated*.” (Clinic B, P 14-Female, >65)

### 3.4 Theme 2: emotional and psychological factors affecting eating habits

Participants described strong emotional reactions following their diabetes diagnosis, including fear, shock, and anxiety. These emotional responses influenced how participants engaged with dietary recommendations and self-management practices.

#### 3.4.1 Emotional shock and fear at diagnosis

Most participants (n = 14) described feelings of fear, shock, or emotional distress following their diabetes diagnosis. For many, the diagnosis was unexpected and associated with fears of severe illness or death.

When asked how they felt after being diagnosed with diabetes, one participant stated:

*“I was shocked because I wasn’t expecting it.”* (Clinic B, P08-Female, 36-50)

Another participant described intense fear following diagnosis:

*“I was very scared. I thought I was going to die.”* (Clinic D, P31-Female, 18-35)

Some participants expressed disbelief or confusion about the diagnosis, particularly when they were not experiencing noticeable symptoms.

“*I did not believe it because I was not feeling any pain*.” (Clinic C, P20-Female, 51-65)

Fear was often heightened among participants who had witnessed family members or community members suffer severe complications or death related to diabetes.

*“My mother also died from diabetes, so it worried me.”* (Clinic B, P05-Female, >65)

One participant linked her fear to concerns about her responsibilities as a parent:

“*I was very troubled. I thought I was going to die. At that time, I had a 2-year-old child. It really shocked me.”* (Clinic B, P13-Female, 51-65)

In some cases, participants perceived diabetes as more threatening than other serious illnesses, reflecting the severity with which the condition was understood.

*“I feared it more than HIV, it felt more dangerous.”* (Clinic C, P17-Male, 51-65)

#### 3.4.2 Processes of acceptance and adjustment

Some of the participants (n = 9) described a gradual process of acceptance and adjustment following their diabetes diagnosis. Although initial reactions were often characterised by fear, stress, or sadness, many participants eventually viewed acceptance as necessary for coping with the condition and making dietary or lifestyle changes.

One participant described moving from emotional distress to acceptance over time:

*“I was stressed, I felt bad, but then I accepted that it’s now part of my life.”* (Clinic A, P01-Female, 51-65)

Some participants indicated that they accepted the diagnosis immediately after being informed.

*“I accepted it immediately when I was told.”* (Clinic D, P30-Female, 51-65)

Others reflected on long-term adjustment to living with diabetes.

“*I accepted it. I got the diagnosis while I was still young, but I have continued to live with it since then*.” (Clinic D, P34-Female, 36-50)

For some participants, acceptance was accompanied by active efforts to modify diet and lifestyle.

*“At first, I didn’t want to accept it, I’ve really tried with my diet.”* (Clinic A, P03-Female, 36-50)

Psychological Burden of Poor Glycaemic Control.

Some participants (n = 12) expressed frustration, distress, and helplessness related to difficulties in controlling their blood glucose levels. Despite taking medication and attempting to follow dietary advice, several participants reported persistent fluctuations in glucose levels, which contributed to emotional strain and reduced confidence in their ability to manage the condition.

One participant expressed confusion and frustration about persistently elevated glucose levels despite adhering to treatment:

*“My sugar levels have gone up and I don’t know why, because I’m taking my treatment.”* (Clinic A, P02-Female, 36-50)

This perceived lack of control contributed to emotional strain and, in some cases, feelings of resignation and self-blame.

*“I try to eat right but it manages me.”* (Clinic A, P04-Female, >84)

Another participant described efforts to restrict certain foods without seeing improvement in glucose control.

“*I try to control it; there are many things I do not eat, but the sugar level does not go down.”* (Clinic D, P33-Female, >65)

In some cases, participants blamed themselves for not having sought more information about diabetes management earlier.

*“I should have researched it myself.”* (Clinic A, P06-Female, 36-50)

### 3.5 Theme 3: structural, economic, and environmental barriers to healthy eating

Participants described multiple structural barriers that limited their ability to follow recommended dietary practices. Financial constraints, household food practices, competing health conditions, and inconsistent dietary guidance emerged as major challenges to healthy eating.

#### 3.5.1 Financial constraints and food insecurity

Almost half of the participants (n = 14) reported that financial difficulties and food insecurity limited their ability to follow recommended diets. Foods perceived as healthy were often described as unaffordable, leading participants to rely on cheaper and more filling staple foods.

One participant explained:

*“The food is expensive; there’s no money at home.”* (Clinic A, P01-Female, 36-50)

Others described eating whatever food was available despite knowing it might not align with dietary recommendations.

*“Sometimes I end up eating whatever is available.”* (Clinic D, P34-Female, 36-50)

Staple foods such as maize meal and rice were commonly prioritised because they were affordable and filling.

*“I try, but I have to buy starch foods (rice, maize meal) because everyone eats them. It becomes difficult for me to have my own separate groceries.”* (Clinic C, P17-Male, 51-65)

#### 3.5.2 Household food practices

Some participants (n = 9) described household food practices as barriers to healthy eating. Shared household meals and limited resources often made it difficult to prepare separate meals for individuals living with diabetes. One participant stated:

*“I do not always follow it because I live with children, it is difficult to cook two separate pots.”* (Clinic C, P26-Female, >65)

#### 3.5.3 Agricultural instability and barriers to home food production

Some participants (n = 8) attempted to supplement household food supplies through home gardening or subsistence farming. However, agricultural instability, financial limitations, and physical fatigue reduced the sustainability of these practices. One participant highlighted the financial barriers associated with farming:

*“There is land available for planting, but the problem is money to buy seeds.”* (Clinic D, P27-Female, 51-65)

Participants also described challenges related to unpredictable crop yields and physical limitations that prevented sustained farming activities.

*“It is money. I try to farm, but sometimes the crops do not do well.”* (Clinic B, P09-Female, 51-65)

*“I used to farm myself, but now I am tired.”* (Clinic D, P32-Female, 51-65)

Competing health conditions and physical limitations. A few participants (n = 5) reported that managing multiple health conditions made it difficult to follow dietary recommendations. The presence of other illnesses, medication interactions, and physical fatigue limited their ability to prepare or access appropriate foods.

One participant described confusion regarding dietary advice while managing another medical condition:

*“I think it means vegetables and starches in moderation. Green vegetable. Now I am confused because I am taking Warfarin for INR, and I was told I must eat orange vegetables. So, I am still confused.”* (Clinic D, P32-Female, 51-65)

#### 3.5.4 Knowledge gaps and conflicting dietary information

Some participants (n = 13) reported uncertainty about healthy eating due to fragmented, inconsistent, or unclear dietary information. Participants expressed confusion regarding what foods they should or should not eat, particularly in the absence of ongoing dietary counselling.

One participant explained that lack of knowledge, rather than financial constraints, was the primary barrier:

*“No, nothing really stops me. I can afford food; the only problem is that I do not know exactly what I should and should not eat. My problem is the lack of knowledge.”* (Clinic B, P06-Female, 51-65)

When participants were asked what they understood by healthy eating, it was frequently described in a narrow manner, largely focused on avoiding sugar and increasing vegetable intake, with some reference to reducing starch and fat. Others expressed uncertainty about the meaning of healthy eating itself.

*“I don’t know what healthy eating really means.”* (Clinic D, P33-Female, >65)

*“Healthy eating means vegetables.”* (Clinic C, P18-Female, 51-65)

*“It means eating vegetables and not eating sweet things.”* (Clinic D, P29-Female, >65)

Some participants additionally mentioned reducing starch and fat intake.

*“It means eating vegetables, not eating too much starch, and removing the skin from meat.”* (Clinic C, P26-Female, >65)

### 3.6 Theme 4: role of healthcare providers and support systems

Participants identified healthcare providers and family members as important influences on diabetes-related dietary practices. While healthcare professionals were generally viewed as trusted sources of information, many participants reported limited dietary counselling, inconsistent health messages, and varying levels of family support.

#### 3.6.1 Trust in healthcare providers

When asked where they thought they should be getting the information from, they all resonated that it should be from healthcare providers.

*“From health professionals.”* (Clinic A, P01-Female, 36-50)

*“From someone who has it and also from the hospital.”* (Clinic D, P35-Male, 18-35)

These responses reflected strong trust in healthcare providers as sources of guidance and support for diabetes management.

#### 3.6.2 Inadequate dietary counselling

Some of the participants (n = 8) reported receiving limited or inadequate dietary counselling at the time of diagnosis. Several participants indicated that they were prescribed medication without receiving detailed explanations about diabetes or practical dietary guidance.

One participant explained:

*“No, I was never given an explanation. The only thing I was told at the hospital is that I have diabetes.”* (Clinic A, P04-Female, >65)

Others described receiving minimal information regarding dietary management.

*“I do not have enough knowledge; I just hear things.”* (Clinic C, P22-Female, 18-35)

Some participants attributed this lack of education to systemic pressures within clinics, such as overcrowding and limited consultation time.

*“I think they couldn’t educate because there were too many people.”* (Clinic A, P01-Female, 36-50)

Fragmented and Inconsistent Health Messages.

Some participants (n = 14) reported receiving fragmented, unclear, or inconsistent dietary advice from healthcare providers. In several cases, guidance focused primarily on food restrictions without providing practical explanations or context-specific recommendations.

One participant stated:

*“I do not have enough knowledge, except for being told what not to eat and not what to eat.”* (Clinic D, P27-Female, 51-65)

Others expressed confusion due to conflicting medical advice, particularly when managing multiple health conditions.

*“I am confused because I am taking Warfarin for INR now, I don’t know what vegetables to eat.”* (Clinic D, P32-Female, 51-65)

These findings suggest that inconsistent messaging contributed to uncertainty regarding appropriate dietary practices.

#### 3.6.3 Family and social support dynamics

Family support emerged as an important influence on dietary behaviour, although participants described both supportive and unsupportive household environments.

Some participants (n = 14) reported receiving encouragement and assistance from family members, particularly with food preparation and meal planning.

*“My child cooks fresh vegetables for me.”* (Clinic D, P33-Female, >65)

However, other participants reported limited or inconsistent support, particularly where family members lacked understanding of diabetes or held negative perceptions of dietary restrictions.

*“My husband says the doctors are starving me.”* (Clinic D, P29-Female, >65)

These findings demonstrate that family and social environments can either support or hinder healthy eating practices among individuals living with diabetes.

Collectively, these findings demonstrate that healthy eating among individuals with diabetes in rural Mnquma is shaped by more than individual knowledge or motivation. Instead, dietary practices are embedded within emotional, social, economic, and health system settings. These findings are discussed in relation to existing literature and theoretical perspectives, considering their implications for diabetes care and nutrition interventions in rural SA.

## 4 Discussion

This study explored perceptions of healthy eating and barriers influencing dietary practices among individuals living with type 2 diabetes mellitus in rural communities of the Mnquma sub-region. The findings indicate that healthy eating in this context is shaped by an interplay of limited and fragmented diabetes knowledge, emotional responses to diagnosis, structural and economic constraints, and gaps in healthcare support. Together, these findings suggest that dietary self-management among people living with diabetes in rural Mnquma cannot be understood solely as an issue of individual choice or motivation, but rather as one embedded within broader social, economic, and health-system realities. Although this study was guided by the TPB, the findings extend beyond individual-level determinants of behaviour. While TPB emphasises attitudes, subjective norms, and perceived behavioural control, participants’ experiences reflected broader influences operating at interpersonal, community, and structural levels. This highlights the relevance of a socioecological perspective in understanding dietary practices within resource-constrained settings.

Participants demonstrated partial and sometimes inconsistent knowledge of diabetes and healthy eating. Many understood diabetes through lived experience, symptoms, or family history rather than through clear biomedical explanations. Healthy eating was often simplified by participants as to eating vegetables, reducing sugar, and avoiding fatty foods or starches. While these perceptions reflect some awareness of dietary recommendations, they also suggest limited understanding of broader dietary concepts such as portion control, meal balance, carbohydrate quality, and the long-term role of diet in glycaemic control. Similar patterns have been documented in South African and other low- and middle-income settings, where limited diabetes knowledge and health literacy contribute to challenges in effective self-management behaviours (20, 21). A national systematic review and meta-analysis which synthesised prevalence data from multiple epidemiological studies conducted across several South African provinces reported that participants often equate healthy eating mainly with vegetable consumption, with limited ability to translate dietary advice into practical and sustainable daily choices within household food environments (13, 22).

In addition, participants’ beliefs about the causes of diabetes included stress, heredity, and sugar intake alone. These findings highlight how cultural interpretations and personal experiences shape health beliefs. Similar findings highlighting the influence of social and contextual factors on perceptions of diabetes have been reported among women diagnosed with gestational diabetes in low-income communities in South Africa (23). Although these explanations may help individuals make sense of illness, they may also divert attention from evidence-based management practices when not addressed through appropriate patient education. Furthermore, limited and inconsistent patient education within healthcare systems in sub-Saharan Africa has been identified as a key barrier to effective diabetes self-management and patient understanding of disease management (24). In the rural Mnquma context, where socioeconomic constraints already limit food choices, inadequate understanding of dietary principles further complicates efforts to adopt and maintain healthy eating behaviours. Similar knowledge-related barriers to effective diabetes management have been identified in a scoping review examining diabetes care in West African primary healthcare settings (25).

An important finding was the strong emotional and psychological response associated with diabetes diagnosis and ongoing disease management. Fear was particularly evident among participants who had witnessed severe diabetes-related complications or death among relatives or community members. These findings are consistent with previous research indicating that diabetes diagnosis is frequently accompanied by psychological distress, including fear, anxiety, and concerns about disease complications (26). In rural and resource-limited settings, such fear may be heightened by limited access to accurate health information and the visibility of negative health outcomes within communities (27).

Participants also described frustration and helplessness when blood glucose levels fluctuated despite medication use or attempts to change their diets. Such experiences undermined self-efficacy and contributed to feelings that diabetes was controlling them rather than the other way around. Evidence suggests that psychological and motivational factors play an important role in influencing adherence to recommended self-care behaviours among individuals living with chronic illnesses (28). Family support also played a significant role in shaping emotional adjustment and dietary behaviour. Participants who reported supportive household environments described assistance with food preparation and encouragement to maintain healthy eating practices. Conversely, lack of understanding or resistance within households contributed to distress and hindered adherence to dietary recommendations. These findings align with previous research indicating that supportive family environments improve emotional wellbeing and diabetes self-management, whereas unsupportive social environments increase diabetes-related distress (29).

Structural and economic barriers were central to participants’ accounts of why healthy eating was difficult to achieve. Participants described multiple intersecting challenges that limited their ability to follow dietary recommendations. Financial insecurity emerged as the most pervasive constraint, with many participants relying on social grants, irregular income, or support from family members. Foods perceived as healthy, such as fruits, vegetables, and lean meats, were often described as unaffordable, while maize meal, rice, and bread were prioritised because they were cheaper and filling for the entire household. These findings reflect broader evidence that financial and environmental barriers often limit access to healthier food options in low-income communities (30). In SA, poverty and food insecurity have been widely documented as significant barriers to dietary adherence among people living with diabetes, particularly in rural communities (31, 32). Household food environments further constrained dietary choices. Many participants lived in shared households where meals were prepared collectively, limiting their ability to follow individualised dietary recommendations. Similar findings have been reported in other rural contexts where shared cultural norms and collective eating practices influence household food consumption and dietary behaviour (33). Although some participants had access to land for subsistence farming, agricultural production was often unstable due to limited resources, lack of agricultural inputs, and declining physical capacity. Evidence suggests that subsistence farming alone may not significantly improve dietary quality without adequate financial and structural support (34). Health system constraints further compounded these challenges. While participants generally trusted healthcare providers as sources of dietary information, limited consultation time, high patient loads, and the absence of dietetic services reduced opportunities for tailored dietary counselling (35, 36). These structural challenges should also be understood within the broader historical context of the EC, where the legacy of apartheid-era policies, including land dispossession and spatial inequalities, continues to shape food systems, livelihoods, and access to resources in rural communities.

Healthcare providers and support systems also played an important role in shaping dietary practices Participants consistently identified healthcare professionals as their primary and most trusted source of diabetes-related information. This reliance reflects the central role of healthcare providers in shaping patients’ understanding of chronic disease management in rural and low-resource settings (37, 38). However, trust in healthcare providers did not necessarily translate into effective dietary self-management. Many participants reported receiving limited, fragmented, or inconsistent dietary counselling. In some cases, advice focused primarily on foods to avoid rather than practical guidance on how to eat within financial and household constraints. Similar challenges have been documented in the South African public health system. High patient loads, limited consultation time, and shortages of dietitians restrict the provision of comprehensive dietary counselling (39, 40).

In the absence of structured professional support, family members often became important sources of informal support. Some participants reported receiving assistance with meal preparation and encouragement from family members, which facilitated healthier eating practices. However, family support was inconsistent and sometimes constrained by limited financial resources or misunderstanding of dietary recommendations. Evidence suggests that family involvement can either support or hinder diabetes self-management depending on household resources, knowledge, and social relationships (41). In addition, some participants reported obtaining information from peers, radio programmes, or other individuals living with diabetes. While such peer learning can be empowering, reliance on informal sources without structured professional guidance may also contribute to misinformation or confusion (42).

In rural Mnquma, difficulties in maintaining healthy eating practices were not primarily due to lack of motivation but rather poverty, food insecurity, limited dietary counselling, competing health conditions, and fragile support systems.

These findings highlight the need for context-sensitive diabetes interventions that move beyond generic dietary advice and instead prioritise sustained nutrition education and practical counselling tailored to local food environments. Strengthening diabetes education within primary healthcare services may improve patients’ understanding of dietary management, while family-inclusive approaches and repeated patient education could enhance long-term adherence to recommended dietary practices. Addressing structural barriers such as food insecurity, poverty, and limited access to dietetic services may also be necessary to support sustainable dietary change among individuals living with diabetes in rural communities.

## 5 Strengths and limitations

A key strength of this study is that it provides in-depth qualitative insight into the lived experiences of individuals living with diabetes in a rural South African context that remains underrepresented in the literature. The use of participant narratives allowed for a deeper understanding of how emotional, social, economic, and health system factors interact to influence dietary behaviour. However, several limitations should be acknowledged. The study was conducted within one sub-region and among participants attending selected primary healthcare clinics, which may limit the transferability of the findings to other contexts. The predominance of female (32 out of 35) participants may also have influenced the perspectives represented. This gender imbalance likely reflects healthcare utilisation patterns in primary care settings, where women are more likely to access health services and participate in health-related research. However, this overrepresentation of female voices may have influenced the findings, particularly in relation to household food preparation, dietary decision-making, and engagement with health services, which are often shaped by gendered roles within households. As a result, male perspectives on diabetes management and dietary practices may be underrepresented and it is possible that important gender-specific barriers such as work-related constraints, health-seeking behaviours, or differing perceptions of illness and diet may not be fully captured in the dataset. This may limit the extent to which the findings reflect the experiences of men living with diabetes in similar settings. Future research should aim to include more balanced gender representation and where possible, explore gender-specific differences in diabetes management behaviours and dietary practices in greater depth.

Despite these limitations, the study provides valuable context-specific insights relevant to diabetes management in rural and resource-constrained settings.

## 6 Conclusion

Healthy eating among individuals living with diabetes in rural Mnquma is influenced by more than individual knowledge or motivation. Participants’ dietary practices were shaped by fragmented understandings of diabetes, emotional distress, poverty, household food dynamics, limited access to affordable healthy foods, and inadequate dietary counselling within the healthcare system. Addressing these challenges highlights the importance of context-sensitive diabetes education, improved dietary counselling within primary healthcare settings, and broader structural interventions that address food insecurity and socioeconomic constraints. Strengthening multidisciplinary diabetes care and integrating family and community support systems may be essential for improving dietary practices and diabetes outcomes in rural communities. While this study does not evaluate interventions, the findings provide important insights that may inform the design of contextually appropriate strategies. These findings should be interpreted within the context of a single sub-region and may not be generalisable to other settings.

## Data Availability

The raw data supporting the conclusions of this article will be made available by the authors, without undue reservation.
