# Supplementary material for: Dietary perceptions and challenges in the management of type 2 diabetes in rural communities of the Eastern Cape, South Africa: a qualitative study
Source: Front Clin Diabetes Healthc. 2026 Jul 8;7:1835644. doi: 10.3389/fcdhc.2026.1835644 (PMC13388071; doi:10.3389/fcdhc.2026.1835644)
Supplement: Supplementary file 1 [file DataSheet1.pdf]

Appendix)APPENDIX A

INTERVIEW GUIDE

Section A: Demographic Information

Age:

Gender:

Marital status:

Level of education:

Household size:

Source of income (e.g., pension, family support, farming, etc.):

Warm-Up Questions

How long have you been living with diabetes?

How has diabetes impacted your daily life?

Core Questions

Can you describe what you understand about diabetes?

What do you believe causes diabetes?

Where do you usually get your information about diabetes (e.g., healthcare professionals, family, community, media)?

Do you feel you have enough knowledge about managing diabetes? Why or why not?

Perceptions of Healthy Eating

What does “healthy eating” mean to you?

How important do you think healthy eating is for managing diabetes?

Are there any foods you’ve been told to avoid or include in your diet?

Barriers to Healthy Eating

What challenges do you face when trying to eat healthily?

Probe: Cost, availability, cultural preferences, time constraints.

How do your family or community members influence your eating habits?

Are there any specific situations or times when you find it particularly hard to follow a healthy diet?

Closing Questions

Is there anything else you’d like to share about your experience with diabetes and healthy eating?

Do you have any suggestions for how healthcare providers or the community can better support people with diabetes?

Closing Remarks

Thank the participant:

"Thank you so much for sharing your time and experiences with me. Your insights are valuable and will help improve our understanding of the challenges people with diabetes face."

"If you have any concerns or additional thoughts after this interview, feel free to contact me at [0646061108]."

"I will now analyse the data, and your responses will contribute to the overall findings of this study."

APPENDIX B

IPHEPHA LEMIBUZO YODLIWANO-NDLEBE

Icandelo A: Ulwazi Malunga Nomntu Othathayo Inxaxheba

Ubudala:

Isini:

Isimo somtshato:

Inqanaba lemfundo:

Inani labantu ekhaya:

Umthombo wemali (umzekelo: ipenshoni, inkxaso yosapho, ukufama, njl.):

Imibuzo Yokuqala (Yokufudumala)

Uthe waziva uneDiyabhetesi nini okokuqala?

Isebenzise njani imihla yakho le meko yeDiyabhetesi?

Imibuzo Ephambili

Ulwazi ngoDiyabhetesi

Ungandichazela ukuba uqonda ntoni ngoDiyabhetesi?

Ucinga ukuba yintoni ebangela iDiyabhetesi?

Ngokuqhelekileyo, ufumana phi ulwazi ngoDiyabhetesi? (umzekelo: amagosa ezempilo, usapho, uluntu, imithombo yeendaba)?

Uva ngathi unolwazi olwaneleyo malunga nendlela yokulawula iDiyabhetesi? Kutheni uthetha njalo?

Ulwazi Nemiqondo Ngokutya Okusempilweni

Kuthetha ukuthini kuwe “ukutya okusempilweni”?

Ulibona libaluleke kangakanani ukutya okusempilweni ekulawuleni iDiyabhetesi?

Kukho na ukutya okuthile okuxelelweyo ukuba kufuneka ukuphephe okanye ukongeze ekutyeni kwakho?

Imiqobo Ngokutya Okusempilweni

Ziziphi iingxaki ojamelana nazo xa uzama ukutya ngendlela enempilo?

Cela iinkcukacha: Ixabiso lokutya, ukufumaneka kwazo, izithethe, ixesha elincinane.

Ungandichazela ngobunzima obukhoyo ukufumana ukutya okusempilweni kummandla wakho?

Usapho okanye uluntu lwakho luchaphazela njani indlela omawutye ngayo?

Kukho na amaxesha athile okanye iimeko ezikwenza kube nzima ukulandela ukutya okusempilweni?

Imibuzo Yokugqibela

Kukho enye into ongathanda ukwabelana ngayo malunga namava akho neDiyabhetesi kunye nokutya okusempilweni?

Unazo na izindululo ngendlela amagosa ezempilo okanye uluntu olunokunceda ngayo abantu abaneDiyabhetesi?

Izivakalisi Zokuvala

Bulela umthathi-nxaxheba:

"Ndiyabulela kakhulu ngokwabelana ngexesha lakho namava akho namhlanje. Ulwazi lwakho lubalulekile kwaye luzakunceda ekuphuculeni ukuqonda kwethu ngemingeni ejongene nabantu abaneDiyabhetesi."

"Ukuba unayo nayiphi na ingcinga okanye umbuzo emva kolu dliwano-ndlebe, zive ukhululekile ukunxibelelana nam kwa [your contact information]."

"Ndiza kuhlalutya idatha kwaye iimpendulo zakho ziya kunceda ekwakheni iziphumo zoluphando."
